# Self-care for maternal and reproductive health in conflict settings: qualitative case study in Nuba Mountains, Sudan

**DOI:** 10.3389/fgwh.2024.1367559

**Published:** 2024-08-29

**Authors:** Sali Hafez, Stella Sadia Samson, Lydia Tanner, Naomi Pendle

**Affiliations:** ^1^ Public Health Policy, London School of Hygiene and Tropical Medicine, London, United Kingdom; ^2^ Research and Evaluations Department, The Research People, London, United Kingdom; ^3^ Department of Social and Policy Sciences, University of Bath, Bath, United Kingdom; ^4^ Centre for Public Authority and International Development, London School of Economics and Political Science, London, United Kingdom

**Keywords:** self-care, maternal health, sexual and reproductive health, humanitarian, conflict, Sudan, communal care

## Abstract

**Introduction:**

Self-care is a critical component of Reproductive, Maternal, and Neonatal Health (RMNH), offering women the knowledge, skills, and autonomy needed for well-being throughout the reproductive cycle. This paper explores the significance of self-care in conflict-affected regions, where access to formal healthcare is limited. Such areas place pregnant women at higher risk due to increased incidents of adverse events during pregnancy and childbirth. Self-care interventions have the potential to enhance access to quality healthcare services.

**Methods:**

Employing a qualitative approach, this study explores RMNH self-care practices among pregnant and post-natal women in the Nuba Mountains. The methods included in-depth semi-structured interviews with 24 participants, comprising pregnant women, recent mothers, and healthcare providers. Purposive sampling was used to capture the experiences of mothers, and thematic analysis identified key patterns and themes in self-care practices. The perspectives of healthcare professionals were included to understand the context of RMNH care in conflict settings.

**Results:**

The study revealed the crucial role of community cohesion in providing emotional and practical support in pregnancy, childbirth, and in the post-natal period. Limited healthcare infrastructure and ongoing conflict-related challenges provided important drivers for self-care practices. A spectrum of self-care interventions ranged from personal hygiene practices to community-supported childbirth and postnatal care. Significant reliance on elder women's wisdom and traditional midwifery was observed, particularly in the absence of formal healthcare facilities. Some women moved to live with family close to the hospital in the weeks before their due dates in order to mitigate the risks of early deliveries, complications, or general insecurity in their home areas.

**Discussion:**

The findings present a compelling narrative of communal self-care, challenging the conventional notion of self-care as solely individualistic. In this setting, the community's role is fundamental, with knowledge sharing and mutual support forming the bedrock of maternal health practices. Elder women, embodying repositories of perceived traditional wisdom, emerge as central figures, guiding pregnant and postpartum women through shared experiences and practices. This collective approach is not merely a cultural characteristic but a necessity born out of the region's limited healthcare infrastructure and ongoing conflict. The study underscores the need to recognize and integrate these communal self-care strategies into broader health interventions.

## 1 Introduction

Self-care is a fundamental aspect of Reproductive, Maternal, and Neonatal health (RMNH), providing women with the necessary knowledge, skills, and agency to maintain their well-being during menstruation, pregnancy, childbirth, and the postpartum period (1). Self-care is “the ability of individuals, families, and communities to promote health, prevent disease, maintain health, and cope with illness and disability with or without a health care provider” (2). This approach aligns with the World Health Organization's (WHO) continuum of care framework for self-care, that centers self-care interventions on individuals and caregivers, while operating within the broader health system level by working closely with healthcare workers and the community (3). In this framing, the purpose of self-care is to increase healthcare access by expanding options for people to safely self-manage particular health needs while maintaining close linkages to community-based and facility-based care.

The effectiveness of various self-care practices, including self-administered oral contraceptives, as well as diet and nutrition, physical activity, and psychological strategies have been reported to be effective and cost-efficient particularly in high-income settings (4, 5). However, in conflict-affected areas where access to healthcare services is severely compromised, self-care practices are even more essential, playing a vital role in supplementing life-saving services for women at reproductive age (6). This particular group exhibits high risks. The evidence indicates a higher incidence of adverse events among mothers exposed to armed conflict, such as miscarriage, stillbirth, prematurity, congenital abnormalities, and premature rupture of membranes (7).

In such settings, self-care interventions have the potential to mitigate risks and maintain the health and safety of both mothers and their newborns (2, 6). By engaging in self-care activities such as proper hygiene practices, self-monitoring of pregnancy symptoms, and adhering to recommended instructions for postpartum care, women can proactively protect themselves from many potential health threats. As a result, there is interest in examining the viability of self-care interventions and protection in conflict settings and understanding how they empower women to take control of their health and provide them with a sense of autonomy and self-determination amidst challenging circumstances (2).

This is the case in the Nuba Mountains, located in the South Kordofan region of Sudan which has suffered from decades of recurrent conflict. Conflict began in June 2011 primarily between the Sudanese government and the Sudan People's Liberation Movement-North (SPLM-N). This prolonged conflict has led to significant humanitarian crises, including displacement, food shortages, and widespread infrastructural damage. Access to basic social services is limited or non-existent for the communities residing in these areas (8). Historical neglect by the Government of Sudan has resulted in significant disruptions in healthcare service delivery, with limited access to contraception, adequate antenatal care, family planning, and emergency obstetric care (9). In the Nuba Mountains, most pregnant women rely on untrained local birth attendants or midwives without access to essential equipment. Complications during labor may require women to embark on dangerous journeys lasting days, including crossing the frontlines, in search of emergency obstetric care (9). Additionally, deeply rooted gender and social norms in South Kordofan contribute to harmful practices, such as child marriage and type-III female genital mutilation (FGM) and Adal circumcision (re-sewing genital parts following childbirth) (10). Infant mortality rates, which vary across the country according to context, gender, education level; income and geography, are highest in regions with a history of war and insecurity (11).

There is insufficient knowledge regarding RMNH self-care interventions in conflict settings (2, 5). This study aims to contribute to filling this gap by examining RMNH self-care and self-protection practices among pregnant and post-natal women in the conflict-affected region of the Nuba Mountains. In humanitarian contexts, self-protection is a critical component of community survival strategies and encompasses actions undertaken by individuals and communities to safeguard themselves from violence, coercion, and deliberate deprivation (12). This concept underscores the agency of affected populations in managing their own safety and security, independent of external aid (12, 13). While self-protection is distinct from self-care, the two are interconnected in this particular context in Nuba. Self-care prioritises health promotion, disease prevention, and health maintenance, whereas self-protection focuses on safeguarding individuals and communities from harm (2, 12).

Using an exploratory qualitative case study approach, the study unpacks the challenges women experience in their pregnancy and delivery journeys and how they make decisions to enhance their resilience and well-being. The study aims to gain a fuller understanding of the practices employed and acknowledges that women's actions encompass not only self-care practices but also a wider spectrum of efforts aimed at safeguarding their physical, emotional, and social health in challenging environments. Moreover, the study examines the access and interaction of women in Nuba to health services and facilities, including antenatal care, family planning and other sexual and reproductive health services. By examining both self-care and self-protection practices, researchers can gain a more comprehensive and nuanced understanding of the multifaceted strategies employed by women, and how they navigate the complex realities of conflict settings. The findings will contribute valuable evidence on self-care and protection in conflict settings, informing policymakers, healthcare providers, and humanitarian organizations in developing strategies and interventions to strengthen self-care tailored to the specific health system and protection challenges encountered in conflict-affected settings similar to Nuba, Sudan.

## 2 Materials and methods

### 2.1 Study area and design

This study used a qualitative case study approach. In-depth semi-structured interviews were used to explore the experiences of pregnant and post-partum women in relation to self-care practices in maternal and reproductive health in the conflict-affected region of Nuba. The qualitative methods allowed for a comprehensive examination of participants’ perspectives, practices, challenges, risks and outcomes, as well as exploring complex and nuanced issues related to maternal and reproductive health. They offered structure as well as the flexibility to gather interviewees’ perspectives on complex issues (14). Despite the many strengths, in-depth interviews are time-consuming methods that require extensive experience in adapting the questions, probing and interpreting verbal and non-verbal communications (15) and providing space for reflection.

The study received ethics approval from the London School of Economics and Political Science Research Ethics Committee (Reference number 1216, under the Safety of Strangers project). The absence of formal ethics committees in South Kordofan (16), coupled with the multifaceted challenges of accessing national committees for our Nuba-based research team, prevented us from obtaining local ethical review.

### 2.2 Sampling and recruitment

Purposive sampling was used to select participants, including women visiting health clinics or midwives, as well as community leaders for their insights. The sampling criteria included women of reproductive age, who were at least 18 years old and who were either currently pregnant as confirmed by a healthcare worker, or who had given birth within the 12 months preceding the date of the interview. Health service providers employed at a healthcare facility frequented by the study participants within the Nuba Mountains were selected for this study.

Data was collected between April 2022 and August 2022, across the Nuba Mountains area, mainly in Kauda, Chawere, Heiban, Kumbur, Luwere, and Iido Payam. A total of 24 in-depth interviews were conducted, comprising three interviews with healthcare service providers and 21 interviews with women of reproductive age, including 11 currently pregnant women and 10 who had delivered within the past 12 months. Two of the three healthcare service providers were male.

Data collection was carried out by three local female researchers under the supervision of an experienced research associate, who provided training on the interview protocol and ethical considerations. The sample size for both participant groups was determined based on data saturation. The sampling and data collection approach aimed to capture the perspectives and explore different experiences among women and service providers.

We relied on networks of midwives and community health workers (CHWs) in Nuba to identify and recruit study participants who met the inclusion criteria. Midwives identified women who were receiving ongoing post-natal care or whom they had assisted in the delivery. CHWs would identify potential participants during their routine door-to-door activities and refer them to the research associate for assignment to a local enumerator.

### 2.3 Data collection

The co-authors collaboratively developed the interview guide, which was subsequently translated into Arabic by co-author. Another co-author verified the Arabic translation for dialect accuracy. The local enumerators received a training session on the interview guide, which included instruction on translating it into the local languages prevalent in the study sites. The majority of interviews were conducted in participants’ homes, specifically in private areas such as backyards, or under a close by tree. As no participant transportation was required, no financial compensation was provided. Three women who were scheduled for interviews declined for family commitments.

The interview protocol included a process for verbal consent. Due to the personal nature of the data, interviews were not recorded. All the interviews were 1-to-1 for confidentiality. Detailed notes were taken during the interviews and additional notes recorded immediately after the interview. Interview transcripts were anonymous. Nubian researchers conducted all the interviews and transcribed them in Arabic or local languages. Later, the research associate translated all the interviews into English. To ensure accuracy, another co-author conducted a quality assurance review by comparing the Arabic and English versions.

### 2.4 Analysis and validation

Thematic analysis was conducted to identify patterns and themes. The analysis was guided by the conceptual framework from the World Health Organization's classification of self-interventions into individual, community, and health system determinants (17). The framework was chosen for its ability to analyse the interplay between care, sexual and reproductive health, and the healthcare system within a nuanced framework of self-care that extends beyond the individual.

**Figure 1:**
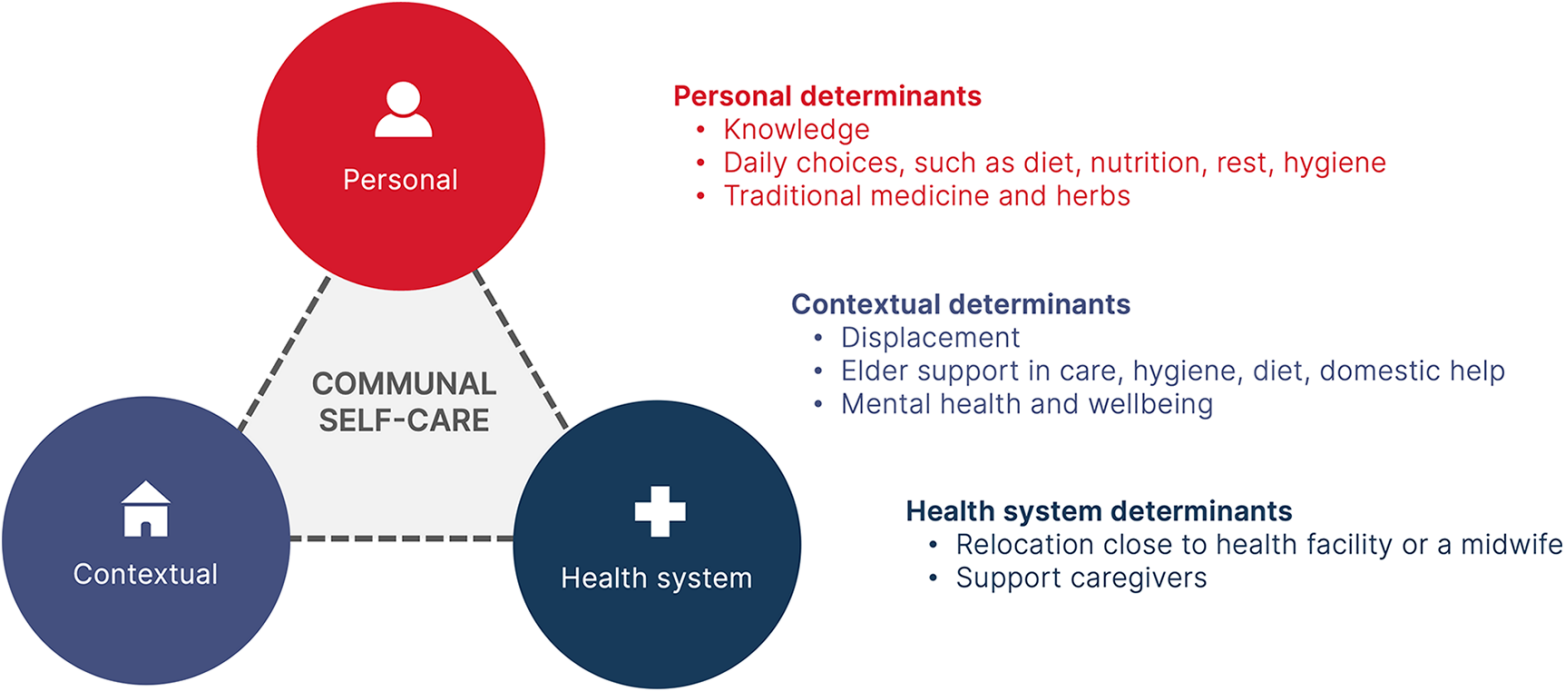
Reported self-care practices in Nuba Mountains. The model is adapted from the WHO Continuum of Care for Self Care Model (14).

All transcripts were coded using digital software. An initial codebook was developed by SS based on the self-care model (Figure 1), with LT and SSS providing subsequent review and validation. Using MAXQDA software (Version 22.2.1) (2022) (18), two members of the team coded all the transcripts, then conducted a review and validation of the coded material. This two-step approach, combined deductive analysis (predetermined codes based on literature and the study framework) and inductive (identifying new themes emerging from the data) approaches, enriched the analysis. The themes that emerged included the emergence of community care structures as a determinant of survival, understanding the drivers of self-care practices, and exploring examples of community practices for self-care.

We ensured the rigour of data collection and analysis through a written study protocol that included the research processes and decisions (19). Researcher bias was reduced through discussions of the emerging findings between team members. Researcher bias was managed through bringing together Findings were validated through a workshop with local enumerators that provided valuable insights into the emerging findings and interpretation. Findings were triangulated by comparing our data to relevant literature.

### 2.5 Participant involvement

Study participants were not involved in the design or planning of the research study. However, we consulted community leaders about the best approaches to sample and conduct the study. The data collection tool was pre-tested with the study participants and specific feedback was obtained on language, sensitivity to some questions and alternative ways to ask them. The study team adapted the tools accordingly. The study participants were involved in participant recruitment by recommending and contacting potential participants to join the study.

### 2.6 Limitations

The study faced several limitations. The translation of qualitative data from Arabic and local languages to English may have introduced nuances and complexities that were difficult to fully capture in translated transcripts. The study is based on a small sample of interviews gathered from women connected to health care facilities in the Nuba Mountains and who were willing to be interviewed. Women who consented to participate may differ in their experiences and perspectives from those who did not have access to the formal health system. Although this may limit the generalisability, reliability, and applicability of findings to other contexts, the in-depth analysis generates valuable hypotheses testable in similar case studies (20).

## 3 Results

The average age of pregnant women was 25 years, while the average age of those who had delivered in the last 12 months was slightly higher at 26 years. Approximately half of the participants had received no formal education or had only completed a basic education.

At the time of the interviews, women had experienced several years of relative peace in Nuba and expressed hopes for dialogue, reconciliation, and rebuilding. However, this was set against a backdrop of decades of insecurity, with threats to physical security, widespread sexual violence, and food insecurity. Almost all the participants had experienced the effects of the conflict including theft, vandalism, threats of violence, loss of loved ones, insufficient food, and trauma. The conflict resulted in poor health infrastructure and neglected investment in and development of the health system capacities. The scarcity of health facilities, combined with inadequate road conditions, posed a significant barrier for pregnant women seeking medical care.

### 3.1 Structures of care and the role of community as a key determinant of survival

The effects of recent waves of conflict were still very present in participants’ responses. Many had experienced the direct consequences of conflict including being exposed to aerial bombardment and random shootings. Mountainous areas were perceived to be safer, and many respondents moved to the mountains to reduce the risk of bombardment. However, this led to scarce food and few livelihood options. Many participants reported loss of properties, houses, land and agricultural products burnt or vandalized, and persistent food insecurity. Several mentioned the prevalence of sexual violence and recruitment of young children into armed groups.

*“I’ve lived here for four years. I was living up in the Mountains for safety reasons during the fighting from 2016 [to] mid 2019”. D04*

*“My daily routine changed during the conflict and insecurity because there was bombardment in my area. We had to run to hide in the bunkers. I had to take the children to the caves early in the morning instead of school and then search for food and cook it in the cave”. D07*

Within this context, being part of a community was seen a crucial determinant of survival, physical wellbeing, and protection during displacement. Knowledge and guidance from other community members and elders allowed women to navigate the conflict. This included how to identify and avoid landmines, how to protect themselves and their families, locations of foxhole's and nearby caves for hiding, road conditions, and how to farm in the mountainous areas. Group farming provided temporary food supplies for some. Community gatherings were an important way of managing stress.

In the absence of a strong health system, many women who participated in this study said that they turned to these same support structures during pregnancy and childbirth. New mothers relied on elder women to share knowledge and advice, including on breastfeeding, nutrition, post-natal care, and caring for the newborn.

### 3.2 Drivers of self-care practices

Some of the study participants shared how knowledge sharing played a pivotal role in women's self-care, particularly given that around half the participants had no or very basic formal education (can read and write). A healthcare worker explained that the scarcity of health facilities and the destruction of radio stations during the conflict further compounded the conflict-induced safety and access challenge by restricting physical access to formal health information for women.

*“We don't have a close health facility, the closest hospital is two days walk and the road is not good”. P4*

Faced with these barriers, the study participants turned to alternative sources of knowledge, placing substantial reliance on perceived traditional wisdom and seeking guidance from experienced midwives, as explained by the study participants. This dynamic illustrates the vital role that knowledge sharing played in bridging gaps in access to critical health information during a time of adversity.

Maintaining good hygiene was seen as important, including bathing themselves with warm water, using traditional backcloths and tree leaves, crafting homemade soap, and following hygiene advice from elder women. Handwashing and maintaining overall cleanliness were emphasized for both pregnant and recently delivered women.

Breastfeeding also served as a motivating factor for women who had recently given birth to uphold a nutritious dietary regimen, reinforcing their overall nutritional status. The dietary practices included the consumption of porridge, soups, fruits, and honey, with the perception that these foods positively contributed to nutritional well-being and favorable outcomes for the newborn. Women often believed that using traditional herbs, along with sesame oil and various tree leaves was also correlated with enhanced immunity for the mother and baby.

### 3.3 Community practices for self-care

Around half of the participants gave birth at home, a decision they made with the guidance of elder women in the community and based upon the challenges in reaching a health facility. Home deliveries were typically supported by midwives, family members, and community elders, who assisted with the delivery process and visited after birth in the postnatal period. The elder women made soap for pregnant mothers and often stayed with them to assist with domestic chores, cook nutritious food, and help take care of the newborn.

*“During my first delivery, I was guided by a lady who was taking care of me. She made sure that I maintained good standard hygiene practices. She ensured that I ate well, on time, and consumed soup and porridge. After giving birth, she supported me to breastfeed my baby. Her presence allowed me to take enough rest while she did domestic work while teaching me how she does so- so when I have another baby then I can follow her practices on my own, and at home”. D7*

Most women relied on elder women for care, as something that they hoped would help to avoid potential pregnancy complications or miscarriage. Miscarriage was perceived to be common among the study participants in Nuba and six out of the twenty-one women that we talked to - either recently delivered or currently pregnant – had experienced either a miscarriage or a neonatal death. None of them identified a prior miscarriage or neonatal death as a risk factor for their current pregnancy.

*“I had three pregnancies before whereby I lost two babies and one child is alive. Not all my pregnancies were successful.” -P04*

Many of the women who had experienced conflict and insecurity struggled with mental health, anxiety, stress and managing uncertainty. Several women expressed how stressful their situation was, unsure how to start their lives over after losing homes, livelihood or moving to a new region. Having a social support structure made many women feel more relaxed, less anxious and assured that they would be supported and protected, particularly if conflict suddenly resumed. Women highlighted the positive impact of community gatherings, chats, entertainment, storytelling, and collective activities on their well-being and distress levels during pregnancy and the postpartum period. Engaging in activities like hair braiding, dancing, and storytelling provided a sense of relief and helped them cope with stress. Faith, prayers, and meditation were also mentioned as important coping mechanisms.

*“Women who [have] just [given] birth need more attention because they are still bleeding and the baby is fragile. Both the mother and the baby need clean and soft cloths, this is why in Nuba there will be a woman caring for the recently delivered woman and the baby. Monitoring her bleeding, supporting hygiene practices, providing clean water and making sure all measures are in line with the best practices for the new mother are done by the appointed caretaker. In most cases, the caretaking duties are done by the mother of the recently delivered woman, [her] mother-in-law or aunt - with the help of young girls fetching water and bringing firewood.” Service provider 1*

### 3.4 Use of traditional medicine

Although most women said they did not use traditional medicine, many combined advice from healthcare professionals with practices learned from their clans or communities. Women were advised to consume porridge, local herbs, honey, leaves, and wild fruits to ensure adequate nutrition during both pregnancy and breastfeeding. Hibiscus was added to water during pregnancy for hydration. Only a few women had access to analgesics for childbirth and traditional herbs were used instead to help manage pain. Half of the study participants from the pregnant and recently delivered women perceived pain to be a natural part of being pregnant or giving birth. Many believed that the use of traditional herbs, sesame oil, and tree leaves contributed to better immunity and improved delivery outcomes.

“*I made sure I have some traditional medicine to use like honey. I spread it on my stomach. I make sure I have some food. Also, I prepared soap and sesame oil”. D08*

“*I prepare myself by staying close to the elderly woman at home who is taking after me during the last trimester of pregnancy. She has good experience taking care of young pregnant women. She makes sure to keep me clean and advises me on how to deal with the pain when it*’*s time for delivery. She also baths me with warm water and prepares for me local perfume, soap and oil for my personal use while monitoring my condition after I give birth to the baby”. D0*

### 3.5 Navigating the wider health system and health infra-structure

The authors observed that the health system in Nuba is minimal, with a small number of health facilities offering maternal and neonatal health services and the hospital being two days walk on a poor road from some of the participant's homes. During the study period, facilities experienced overcrowding and stretched resources, compromising the quality of care provided. While there were established referral pathways, poor transport and bad roads limited their effectiveness.

*“I do attend my maternal health information session at the Luwere hospital. I saw pregnant women suffering, in the hospital because of the high demand for care and long queues for pregnant women. The facility cannot accommodate all that. therefore is a bit heavy to go there. However, I felt uncomfortable and went to the hospital where I was given medication”. P5*

Pregnant women sought basic antenatal care services from the health facilities, including access to supplements like iron. Some women moved to live with family close to the hospital in the weeks before their due dates in order to mitigate the risks of early deliveries, complications, or general insecurity in their home areas. However, long distances discouraged many women from seeking professional care.

## 4 Discussion

The Nuba Mountains form a unique backdrop for understanding the importance of self-care and community support for women during and after pregnancy. The region is notable for the diverse protection risks that its inhabitants have faced, the scarcity of international protection, and the remarkable levels of self-reliance among the population living there that have been documented (21). Previous research illustrated that Nubian women relied heavily on strong communal unity and cooperation to meet their physical and emotional needs during conflict (16). A wide range of self-protection mechanisms were documented ranging from making extensive medical and care use of trees, local herbs and plants, to fostering communal unity and belonging, to moving to the mountains or working at night to increase physical safety. During conflict, for example, community institutions such as schools, mosques, and churches were sometimes moved to caves or forests to enhance safety during bombings (22).

Our findings reinforce the importance of community structures for women navigating pregnancy and childbirth amidst limited formal healthcare. Self-care, in this context, is not individualistic but is communal involving collective activities, knowledge sharing, living with family and much more. Knowledge sharing and mutual support form the bedrock of Nubian maternal health practices. For example, elder women emerge as key figures in safeguarding the well-being of families, offering practical assistance and being repositories of traditional knowledge on natural resources crucial for women's health and well-being. This included the use of traditional herbs, handmade soaps, and other natural products for self-care during pregnancy and post-natal periods. Women sometimes elected to move closer to family, elders, or healthcare facilities as they prepared for childbirth and the early weeks of the post-partum period.

In our interviews, local knowledge systems and traditional cultural practices were perceived to promote Sexual and Reproductive Health (SRH) well-being. A similar 2024 study on SRH self-care practices, and the enablers and barriers of these practices among reproductive-age women in Nepal echoed these findings (23). However, few Nuban women have access to scientific knowledge on the evidence for these practices. While some traditional/community-based practices are beneficial, others may be harmful- and the literature highlights the influence of community elders in driving Female Genital Mutilation (FGM) in Sudan, for example (24). A nuanced approach is required, acknowledging both the potential and the risks associated with cultural and traditional practices.

The psychological needs of war-affected individuals are just as critical as their physical needs. Women have faced prolonged insecurity, sexual violence and rape as a weapon of war, and a scarcity of formal protection. Nubian women who had experienced conflict and insecurity reported struggles with mental health, anxiety, stress and managing uncertainty. Support and comfort within the community have previously been highlighted as crucial in helping individuals cope with these experiences (22). During pregnancy and post-natal period many women explained how community chats, songs, entertainment, storytelling and collective activities positively enhanced their well-being and distress.

Local organisations can help strengthen communal self-care practices. In the Nuba Mountains, there is an impressive history of local NGOs supporting self-care initiatives. During the last period of conflict, for example, a local NGO called NRRDO provided four-day training sessions for volunteers who then disseminated the knowledge within their respective communities. This included instructions on how to respond and protect oneself from imminent bombing threats (22). A 2014 evaluation showed that 80% of 640 randomly selected households knew about all the protection messages including digging foxholes, hiding in caves, family budgeting, food storage, health, sanitation and first aid (22). Such structures could provide a productive way of reinforcing SRH self-care.

The already stretched health facilities in the Nuba Mountains did not offer any interventions designed to promote self-care such as pregnancy self-testing, self-monitoring of blood glucose during pregnancy, or post-natal contraception. However, these interventions could encourage stronger individual-level and facility-level self-care and contribute to the quality of care.

Collective self-care approaches are not merely a cultural characteristic but are a necessity born out of the region's limited infrastructure and ongoing conflict. They are not sufficient on their own. During pregnancy and childbirth, women living in conflict settings face heightened risks due to factors such as FGM, previous sexual violence, poor nutrition and other unaddressed health needs. Moreover, pregnant women in Sudan may face clinical complications, such as hypertension, preeclampsia, eclampsia, obstructed labor, haemorrhage, sepsis, and ruptured uterus (25).

Community-based self-care should supplement and not replace formal healthcare. Our study underscores the urgent need for comprehensive, culturally appropriate RMNH services, and substantial investment in building of health infrastructure in the Nuba Mountains. Echoing the study participants, we emphasize the need for well-equipped, geographically accessible hospitals staffed by skilled healthcare providers. The responsibility for care should sit with national actors, humanitarian organisations and health professionals, and not continue to fall on the community.

Nevertheless, the existing collective protection mechanisms established in regions like the Nuba Mountains underline the value of recognising and integrating communal self-care initiatives into broader health interventions. This is particularly pivotal for maternal care, considering the disproportionate impact of sexual and reproductive health conditions on the well-being of women in conflict settings. The WHO's guidelines, including crucial elements like pregnancy self-testing, offer an approach to empowering women with the knowledge and tools they need to take charge of their health. By fortifying these efforts, humanitarians could forge a path towards safer, more empowered maternal care in challenging environments, ensuring better outcomes for both mothers and their newborns (26).

## Data Availability

The original contributions presented in the study are included in the article/Supplementary Material, further inquiries can be directed to the corresponding author.
